# Use of a Cellulase from *Trichoderma reesei* as an Adjuvant for *Enterococcus faecalis* Biofilm Disruption in Combination with Antibiotics as an Alternative Treatment in Secondary Endodontic Infection

**DOI:** 10.3390/pharmaceutics15031010

**Published:** 2023-03-21

**Authors:** Selene Velázquez-Moreno, Ana Maria González-Amaro, Antonio Aragón-Piña, Lluvia Itzel López-López, Roberto Sánchez-Sánchez, Mario Alberto Pérez-Díaz, Ricardo Oliva Rodríguez, Ana C. Lorenzo-Leal, Omar González-Ortega, Fidel Martinez-Gutierrez, Horacio Bach

**Affiliations:** 1 Microbiology Laboratory, School of Chemical Sciences, Autonomous University of San Luis Potosí, San Luis Potosí 78300, Mexico; 2 Endodontics Postgraduate Program, School of Dentistry, Autonomous University of San Luis Potosí, San Luis Potosí 78300, Mexico; 3 Electronic Microscopy Laboratory, Institute of Metallurgy, Autonomous University of San Luis Potosí, San Luis Potosí 78300, Mexico; 4 Institute of Desert Zones, Autonomous University of San Luis Potosí, San Luis Potosí 78300, Mexico; 5 National Institute of Rehabilitation, Mexico City 14389, Mexico; 6 Biomembranes Laboratory, National School of Biological Sciences, National Polytechnic Institute, Mexico City 07738, Mexico; 7 Division of Infectious Diseases, Department of Medicine, University of British Columbia, Vancouver, BC V6T 1Z4, Canada; 8 Bioseparations Laboratory, School of Chemical Sciences, Autonomous University of San Luis Potosí, San Luis Potosí 78300, Mexico; 9 Center for Research in Health Sciences and Biomedicine, Autonomous University of San Luis Potosí, San Luis Potosí 78300, Mexico; 10 Laboratorio de Antimicrobianos Biopelículas y Microbiota, Facultad de Ciencias Químicas, Autonomous University of San Luis Potosí, San Luis Potosí 78210, Mexico

**Keywords:** extracellular polymeric substances, bioreactor, endodontics, enzyme, root canal treatment, biofilm, *Enterococcus faecalis*, cellulase, inflammatory response

## Abstract

Apical periodontitis is an inflammation leading to the injury and destruction of periradicular tissues. It is a sequence of events that starts from root canal infection, endodontic treatment, caries, or other dental interventions. *Enterococcus faecalis* is a ubiquitous oral pathogen that is challenging to eradicate because of biofilm formation during tooth infection. This study evaluated a hydrolase (CEL) from the fungus *Trichoderma reesei* combined with amoxicillin/clavulanic acid as a treatment against a clinical *E. faecalis* strain. Electron microscopy was used to visualize the structure modification of the extracellular polymeric substances. Biofilms were developed on human dental apices using standardized bioreactors to evaluate the antibiofilm activity of the treatment. Calcein and ethidium homodimer assays were used to evaluate the cytotoxic activity in human fibroblasts. In contrast, the human-derived monocytic cell line (THP-1) was used to evaluate the immunological response of CEL. In addition, the secretion of the pro-inflammatory cytokines IL-6 and TNF-α and the anti-inflammatory cytokine IL-10 were measured by ELISA. The results demonstrated that CEL did not induce the secretion of IL-6 and TNF-α when compared with lipopolysaccharide used as a positive control. Furthermore, the treatment combining CEL with amoxicillin/clavulanic acid showed excellent antibiofilm activity, with a 91.4% reduction in CFU on apical biofilms and a 97.6% reduction in the microcolonies. The results of this study could be used to develop a treatment to help eradicate persistent *E. faecalis* in apical periodontitis.

## 1. Introduction

Apical periodontitis is an acute inflammation with lesions that can progress to a chronic state, injuring and destroying periradicular tissues. It occurs in the apex of the tooth root and is mainly caused by bacterial infection. Apical periodontitis starts from dental pulp damage due to root canal infection, endodontic treatment, caries, traumas, cracks, or other dental interventions [1]. 

According to a systematic analysis of the global burden of 328 diseases, permanent caries and periodontal diseases ranked in first position and eleventh position, respectively, among the most prevalent human infections worldwide. Periodontal diseases affect 750 million people globally [2]. 

Biofilms are complex microbial structures known as extracellular polymeric substances (EPS). These structures defend microorganisms against environmental insults, including antibiotics, dryness, detergents, etc. [3,4]. These biopolymers include polysaccharides, proteins, nucleotides, and lipids, which play a central role in the adhesion and survival of bacteria. EPS anchor microorganisms to the surface, maintaining their structural integrity [5]. 

The presence of biofilms during pathogen infections constitutes a challenge for treating, healing, and establishing a healthy oral environment. In this regard, it has been reported that approximately 20% of treatments fail because of biofilms [6]. Endodontic infection treatment is based on broad-spectrum antimicrobials combined with sodium hypochlorite (NaOCl) [6]. 

After root canal treatment, proper disinfection is necessary to create a microenvironment that facilitates repopulation of the cleaned areas with host cells derived from the apical papilla stem cells (SCAP) [7]. However, the use of instrumentation to treat root canals is not recommended in necrotic immature permanent teeth because it might weaken the dentin walls. Aligned with this issue, accepted treatments such as NaOCl irrigation also possess a high potential for organic matter degradation, compromising SCAP integrity [8].

There is a constant search for clinical strategies for decontaminating the root canal system without impeding the fixation, proliferation, and differentiation of SCAP. Some alternatives to endodontic irrigants have been explored, such as herbal and natural products, which could have good biocompatibility compared with chemical compounds [6]. Other biomolecules, such as enzymes and peptides, have been proposed for biofilm disintegration [9,10,11]. For instance, studies of fungal proteins, classified as glycoside hydrolases, could digest and break down polysaccharides [12,13,14]. One example of these enzymes is the glycoside hydrolase produced by the fungus *Trichoderma reesei*. However, this particular enzyme has not been studied for its activity against *E. faecalis* biofilms. *E. faecalis* is a pathogen commonly isolated from persistent secondary infections associated with failure of root canal treatment [15]. It is difficult to eradicate [16], mainly because of its high adaptability for survival under unfavorable environmental conditions [2], and its high capability for producing complex and intricate biofilms [17].

**Figure 1:**
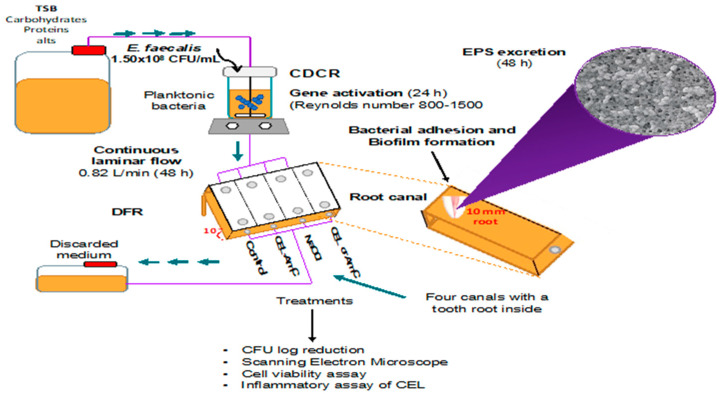
Scheme representing a Center for Disease Control reactor. CDCR, CDC reactor; DFR, drip flow reactor; TSB, trypticase soy broth; EPS, extracellular polymeric substances; CEL, hydrolase; AmC, amoxicillin/clavulanic acid; CFU, colony-forming units.

Bioreactors have been designed to control experimental parameters such as nutrients, flow rate, hydrodynamic forces, and cell density. They are considered valuable tools for studying microbial biofilms in vitro. A drip flow reactor (DFR) (BioSurface Technologies, Corp., Bozeman, MT, USA) consists of channels containing an active substrate in which the biofilm is grown under a continuous laminar flow [18,19]. The reactor allows microorganisms to establish EPS on desired structures, such as the dental root apex (Figure 1). 

The present study tested the antibacterial activity of a *T. reesei* hydrolase (CEL) combined with an antibiotic mixture of amoxicillin/clavulanic acid (AmC) against *E. faecalis*. To determine if this combination can be developed as a potential therapy, its cytotoxicity and its inflammatory response were tested on human fibroblasts and the human-derived monocytic cell line (THP-1), respectively. 

## 2. Materials and Methods

### 2.1. Patients and Strains

The protocol for sample collection was approved by the Faculty of Dentistry’s Investigation Ethics Committee (CEI-FE-005-1-018) and the Ethics Committee of the Autonomous University of San Luis Potosi (CONBIOETICA-24CEI-003-20190726). The study included a clinical *E. faecalis* strain isolated from a central venous catheter (E.F MD), a reference *E. faecalis* strain (ATCC 29219), and nineteen clinical strains of *E. faecalis* (E.F M1-19) isolated from patients who experienced failed endodontic treatments due to having apical periodontitis. The nineteen strains were identified using API 20 Strep (Biomerieux, Craponne, France), and the sensitivity profile of the planktonic bacteria was carried out using the Kirby–Bauer disk diffusion method (Supplementary Tables S1 and S2) [20].

### 2.2. Biofilm Plate Assay

Bacterial strains were grown overnight at 37 °C in trypticase soy broth (TSB) (Becton Dickinson, Mexico City, Mexico). The inoculum was standardized to an optical density (O.D.) of 0.08 at 625 nm. A 96-well culture plate was filled with 180 μL of fresh TSB and 20 μL of the inoculum (approximately 1.5 × 10^8^ CFU/mL). In addition to the nineteen endodontic clinical strains, two controls were used: a clinical isolate from a central venous catheter as the positive control and a saline solution as the negative control. The plates were incubated for 48 h at 37 °C and 110 rpm. The culture media were discarded, and the wells were gently washed three times with 200 μL of PBS to remove non-adherent bacteria. The plates were air-dried and stained with 0.1% crystal violet solution (HYCEL, Zapopan, Mexico) for 10 min. The excess stain was removed by washing thrice with 200 μL of sterile distilled water. The plates were dried for 10 min at 60 °C, and 200 μL of absolute alcohol (J. T. Baker, Phillipsburg, NJ, USA) was added to each well for dye solubilization. The O.D. of the samples was measured at 570 nm with a Multiskan FC microplate photometer (ThermoFisher Scientific, Waltham, MA, USA). All tests were performed in triplicate. The spectrophotometric assessment of the biofilm formation was done according to published protocols [21]. The biofilm formation categories were defined as non-producers (O.D. value < 0.086), moderate biofilm producers (O.D. values ranging between 0.086 and 0.258), and high biofilm producers (O.D. value > 0.258) [21].

### 2.3. Saccharide Production and Detection Assay

Three hydroxyapatite coupons (BioSurface Technologies Corp., Bozeman, MT, USA) were placed in a 24-well polystyrene tissue culture plate. Then, 150 μL of inoculum (O.D. measured at 625 nm, approximately 1.5 × 10^8^ CFU/mL) and 1350 μL of TSB were added per well. The plates were incubated for 5 days at 37 °C and 110 rpm, changing the TSB broth every 48 h, and subsequently dried for 1 day at 50 °C. The samples were analyzed using a Nicolet 6700/Smart iTRro Fourier-transform infrared spectrometer with attenuated total reflection (FTIR-ATR) (ThermoFisher, Waltham, MA, USA) from 4000 to 700 cm^−1^. The results showed that the *E. faecalis* strain M4 (M4) produced the highest polysaccharide content; therefore, it was chosen for the remaining experiments. The *E. faecalis* strain ATCC 29212 was used in its planktonic state as a negative control for EPS production.

### 2.4. Periapical Biofilm Production

Anterior human teeth with roots over 13 mm and a maximum curvature of 20° were selected. The roots were standardized using a double-sided diamond disc (10 mm long). They were filled with 10 to 25 K-files (Dentsply Sirona, York, PA, USA) to permeabilize the conduction and allow medium flow until the apex was open. The samples were subjected to ultrasound cleaning (BioSonic UC50, Coltene/Whaledent Inc., Cuyahoga Falls, OH, USA) with immersion protocols using 17% EDTA (J.T. Baker) and 5.25% NaOCl solutions, followed by sterilization at 121 °C and 15 psi for 20 min [19]. 

In this study, the biofilms were generated in a DFR, which allowed biofilm growth at the air/liquid interface [22]. A Center for Control Disease (CDC) reactor (BioSurface Technologies) was assembled as per the ASTM E2647-13. The CDC reactor was filled with 800 mL of TSB and inoculated with 1 mL of the M4 strain (O.D. 0.08 at 625 nm, approximately 1.5 × 10^8^ CFU/mL) at room temperature (25 ± 1 °C). Continuous stirring was carried out for 1 h, keeping the Reynolds number in the 800–1500 range. Afterward, the CDC reactor was attached to a sterile TSB supply and to the DFR, which included four channels at a 10° angle with one dental apex each. The bacterial flow of TSB was maintained at room temperature for 24 h using a continuous laminar flow rate of 0.82 mL/min [23]. Biofilms were developed using constant flow over twenty-three dental apices as substrates.

### 2.5. Enzymatic Biofilm Hydrolysis

#### 2.5.1. Hydrolysis Conditions

A hydrolase (CEL, Celluclast R, Novozymes, Denmark) was used in this study. The activity of CEL toward *E. faecalis* biofilms is shown in Figure S1. Different concentrations, pH values, and contact times were tested to determine the appropriate conditions for enzymatic activity. Crystalline cellulose (Sigmacell 20 µm, Sigma-Aldrich, St. Louis, MO, USA) was used as the CEL substrate. A typical reaction was performed at 37 °C for 120 min. Three pH values (5, 7, and 8) and two CEL concentrations (10 and 100 U/mL) were evaluated. Following hydrolysis, the absence of reducing sugars in the reagents was tested using the 3,5-dinitro salicylic acid (DNS) method after centrifugation. In a typical reaction, 50 μL of supernatant was mixed with 450 μL of deionized water and 500 μL of 1% DNS reagent [24]. The reaction was carried out at 90 °C for 15 min until the development of a red-brown coloration was observed. Then, 167 μL of 40% K tartrate·4 H_2_O solution (Sigma-Aldrich) was added to stabilize the coloration. After cooling the reactions at room temperature, the absorbance was recorded with a spectrophotometer at 575 nm. CEL and crystalline cellulose were used as negative controls, and experiments were performed in triplicate.

#### 2.5.2. Periapical Biofilm Hydrolysis

Three biofilms from three dental apices obtained from the CDC reactor were exposed to 1 mL of CEL at 100 U/mL at pH 5 for 1 min by immersion (ten times). The total volume was recovered to determine the concentration of reducing sugars using the DNS method previously mentioned. A 100 mg/dL glucose standard solution (Spinreact, Girona, Spain) was used as a positive control. Experiments were performed in triplicate. 

### 2.6. Antibiofilm Assays

#### 2.6.1. Microcolony Biofilm Assay

The M4 strain was grown on biofilm membranes (0.2 µm nylon, ThermoScientific, Rochester, NY, USA) by depositing 10 μL of the bacterial suspension (O.D. λ = 600 nm equivalent to 0.5 on the McFarland scale) and incubating for 24 h at 37 °C. Subsequently, the biofilm membranes were divided into five groups: untreated control, CEL, AmC, CEL-AmC, and NaOCl. The membrane biofilms were exposed to the different treatments by immersion in 24-well plates for 10 min. The antibiofilm activity of the treatments was evaluated by serial dilutions after vortexing the membranes in 0.85% NaCl, using TSB supplemented with 1.5% agar and incubation at 37 °C for 24 h. The following formula was used to express results as a colony-forming-unit (CFU) percentage = P = (1 − 1^−L^) × 100, where P is the result of the CFU decrease in percentage, and L is the result of the log reduction of CFU.

#### 2.6.2. Periapical Biofilm Assay

Twenty biofilms were developed on dental apices in the reactor at a 1 mL/min flow rate. They were randomly divided into four groups (n = 5): Group I: 10 mL of 0.85% saline solution (negative control), Group II: 10 mL of 2.25% NaOCl solution (positive control), Group III: 10 mL of AmC solution, and Group IV: 10 mL of CEL-AmC solution. The biofilms were irrigated by means of a blunt-ended 0.40 mm gauge needle (Endo-Eze, Ultradent, South Jordan, UT, USA). A soft ultrasonic device (tip 15) was used at frequencies of 20–30 kHz at 1 mm from the apical foramen during the final minute of irrigation in each group [25,26]. The antimicrobial activity was evaluated on three treated apices per group (as previously described). Two of the treated apices per group were prepared for scanning electron microscopy (SEM) (JEOL JSM-6610 LV, JAPAN) to evaluate the development of *E. faecalis* biofilms. Additionally, an experimental device was developed using a continuous DFS to form biofilms under anaerobic conditions. The inoculum was replaced every 24 h with fresh TSB for 10 days. Gram staining was performed daily to evaluate the development of contaminant strains. SEM also evaluated the biofilms developed under this system. This analysis showed mushroom-shaped structures corresponding to a mature *E. faecalis* biofilm. 

### 2.7. Cell Viability Assay

The calcein and ethidium homodimer (EthD-1) assays were performed using the mammalian cells live/dead viability/cytotoxicity kit (ThermoScientific, Rochester, NY, USA). Dermal fibroblasts were obtained from donated skin from aesthetic surgeries, each donor having previously signed a letter of consent. Cells were seeded (1.5 × 10^3^ cells per well) in 96-well culture plates, which were incubated at 37 °C supplemented with 5% CO_2_. Afterward, the cells were subjected to the following treatments for 24 h: untreated (PBS) as a negative control, CEL, AmC, CEL-AmC, and NaOCl (positive control), and according to the conditions predetermined earlier. Culture media were replaced with fresh TSB supplemented with 5 mM calcein and 5 mM EthD-1. The plates were incubated for 1 h, and the cells were imaged using an epifluorescence microscope (AxioVert A1, Zeiss, Oberkochen, Germany). Live and dead cells were counted using ImageJ software. The percentages of live cells were calculated using GraphPad Prism 6.0.

### 2.8. Inflammatory Assay of CEL

The inflammatory response of CEL was tested using the human-derived monocytic cell line THP-1 (TIB-202, ATCC) following a methodology previously published [27]. Cells were dispensed in 96-well plates at a final concentration of 1.0 × 10^5^ cells/well after supplementation of phorbol 12-myristate 13-acetate at 40 ng/mL (Sigma-Aldrich). Cells treated with 1 µg/mL of lipopolysaccharide (LPS) from *E. coli* (Sigma-Aldrich) and 1 mM of prednisolone (Sigma-Aldrich) were used as positive controls. Untreated cells were used as a negative control. The experiments were carried out in triplicate, and CEL concentrations of 25, 50, and 100 U/mL were used. 

### 2.9. Statistical Analysis

The differences between groups were analyzed using an ANOVA or the Kruskal–Wallis and Mann–Whitney U tests. The tests were performed at a 95% confidence level (*p* = 0.05). All statistical analyses were performed using SPSS 23.0 software (SPSS Inc., Chicago, IL, USA) and GraphPad Prism 6.0.

## 3. Results

### 3.1. Selection of a Clinical Strain of E. faecalis

**Figure 2:**
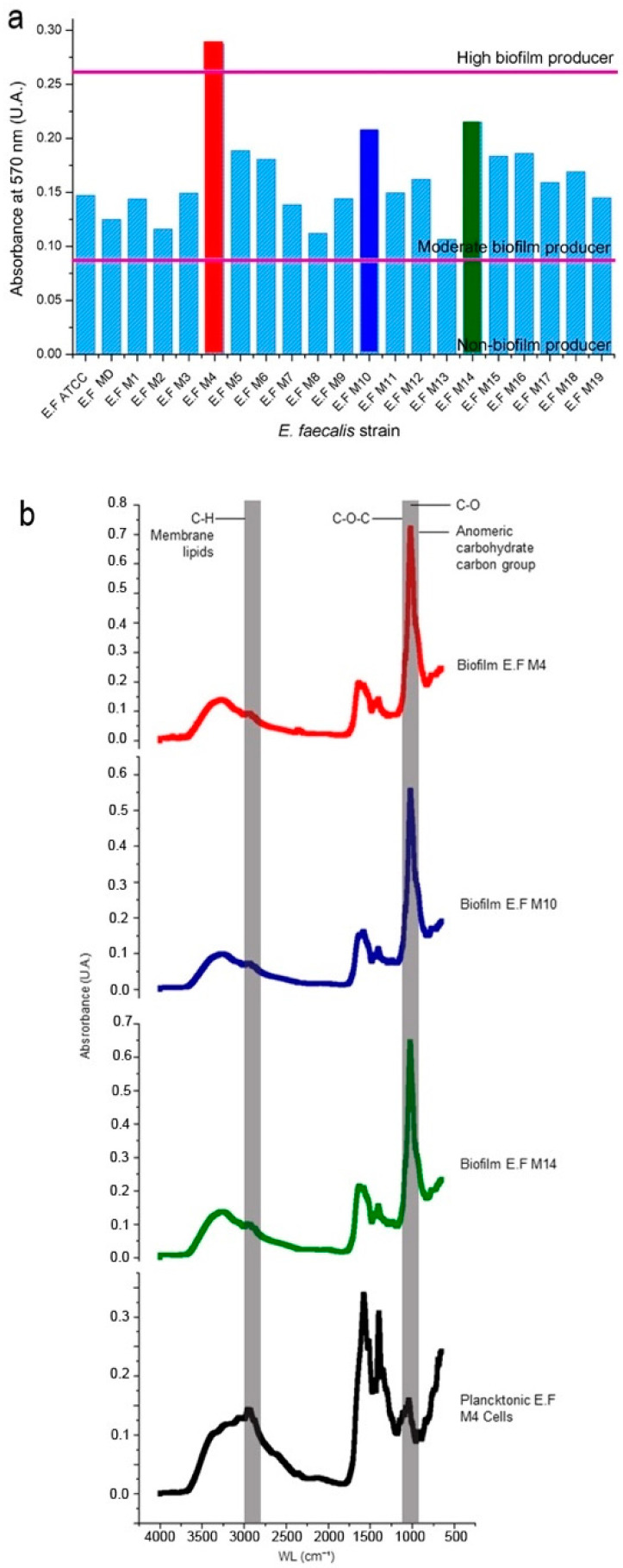
Biofilm production by *E. faecalis* strains and infrared spectra analysis of the biofilms produced by the selected strains. (**a**) Levels of biofilm production according to the absorbance recorded from the analysis of *E. faecalis* strains, the horizontal lines differentiating the three levels of production. (**b**) The characteristic bands of saccharides (gray areas) between 4000–800 cm^−1^ are shown. The difference in vibrations between the spectra for biofilms and planktonic cells (non-biofilm production control) is evident mainly in the vibration bands of C-O, C-O-C, carbohydrate anomeric carbon, and C-H from membrane lipids. E.F ATCC, *E. faecalis* ATCC strain; E.F MD, *E. faecalis* isolated from a medical device (as a positive control for EPS production); E.F M1-19, *E. faecalis* clinical strains.

The resistance profile of nineteen planktonic strains was carried out by the Kirby–Bauer disk diffusion method, which showed that all the strains were sensible to AmC and vancomycin. After a preliminary screening of the strains, the M4 strain showed the highest biofilm production, followed by M10 and M14 (moderate producer strains, Figure 2a). These three strains were selected for the next steps of the study. 

The production of biofilms by these strains showed similar patterns when the biofilms were analyzed by infrared spectroscopy (Figure 2b). The results showed broad absorption bands around 3250 cm^−1^ (O-H stretching) and 1000 cm^−1^ (C-O and C-O-C stretching), suggesting the presence of absorption bands for saccharides at 1640 cm^−1^ (amide I: C=O stretching) and for proteins at 1536 cm^−1^ (amide II: N-H bending). For the planktonic spectrum of M4, the absorption band at 3250 cm^−1^ overlapped with a strong absorption band at 2950 cm^−1^ for lipids (C-H stretching of CH_2_) and at 1640 and 1536 cm^−1^ for proteins [28]. The biofilm and planktonic spectra analyses showed important differences, especially in the absorption bands for proteins, which were decreased in the biofilms. In contrast, the saccharide absorption bands were reduced in the planktonic state. In summary, the infrared analyses confirm saccharides as the main components of the extracellular polymeric matrix of the biofilm.

### 3.2. Optimal Hydrolysis Conditions

Different conditions were tested to determine the optimal enzymatic activity of CEL on cellulose (Table 1). The optimal hydrolysis was obtained at pH 5, with an average glucose release of 0.79 mg/mL at a concentration of 100 U/mL for 10 min. 

### 3.3. Antibiofilm Activity Assays

**Figure 3:**
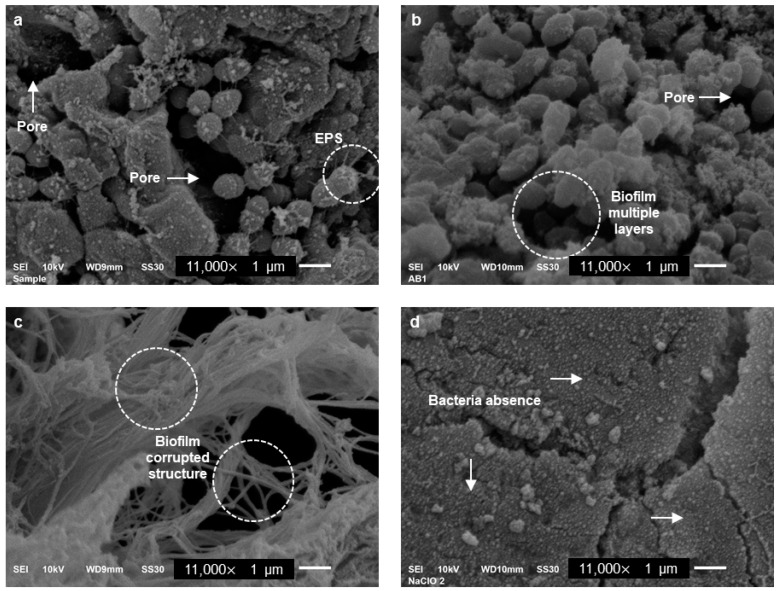
Presence of periapical biofilms after irrigation. The micrographs show the biofilms according to the treatment. (**a**) Untreated negative control group. (**b**) AmC treated group. (**c**) CEL-AmC treated group. (**d**) NaOCl treated group. EPS, extracellular polymeric substances; AmC, amoxicillin/clavulanic acid; CEL, cellulase; CEL-AmC, a combination of CEL/amoxicillin/clavulanic acid.

After treating the biofilm colonies with CEL using the predetermined condition (100 U/mL, pH set to 5, and 10 min of incubation), a statistically significant difference was measured when the CEL/AmC treatment was used (Table 2). The development of a mature biofilm in the periapex is shown in Figure 3a.

In the AmC irrigated group, a 50% reduction in the CFU counting was measured (Figure 3b), showing an EPS coating with multiple layers. For the group irrigated with the CEL-AmC (50 U/mL, 1000 µg/mL, and 250 µg/mL, respectively) treatment, a CFU reduction > 90% was measured, with a concomitant disintegration of EPS and the absence of microorganisms (Figure 3c). Lastly, the NaOCl treatment showed a CFU reduction of 100% (positive control, Figure 3d). 

Based on the ANOVA, the Tukey analysis, and the Bonferroni post hoc test, the groups irrigated with NaOCl and CEL-AmC were statistically significant compared with the control, indicating that the enzyme–antibiotic combination was effective against the *E. faecalis* periapical biofilm. The initial area of the analyzed dental apex is shown in Figure S2.

### 3.4. Cell Viability Assay

**Figure 4:**
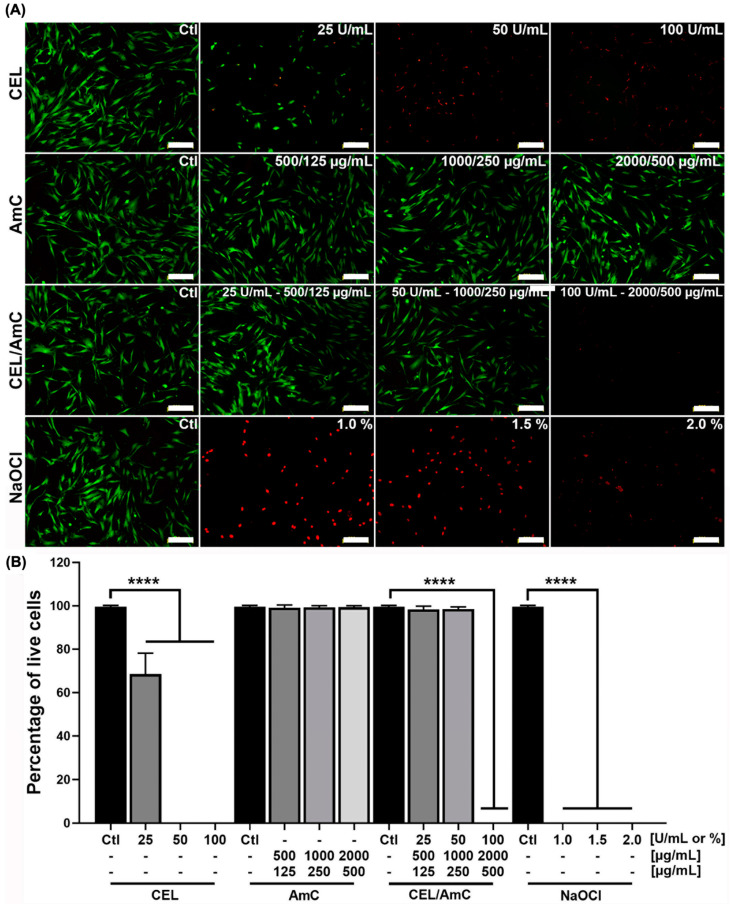
Viability of dermal fibroblasts. (**A**) Cells were exposed to different treatments and incubated with calcein (positive in green) and ethidium bromide (dead in red). (**B**) Viability results are expressed as a percentage of live cells compared with the control group (PBS). CEL, cellulase; AmC, amoxicillin/clavulanic acid; CEL-AmC; cellulase combined with amoxicillin/clavulanic acid; NaOCl, sodium hypochlorite. ANOVA was performed with Dunnett’s post hoc test. **** *p*-value < 0.00001. Scale bars = 200 μm.

The cytotoxicity analysis of CEL, antibiotics, or a combination of both was performed using dermal fibroblasts. CEL at 25 U/mL rendered viability of 72%, with no cell survival at higher enzyme concentrations (Figure 4). The antibiotic treatments showed viabilities >97%, similar to the treatment with CEL/AmC (CEL at 50 U/mL and AmC at 1000 and 250 µg/mL, respectively), which resulted in 98% of live cells. However, higher CEL concentrations in the combined treatment were cytotoxic. In the case of the NaOCl treatment, no live cells were found, as expected (Figure 4). The results also showed that 50 U/mL of CEL was toxic to fibroblasts, but this cytotoxicity was not observed when CEL was combined with AmC. We hypothesized that this lack of cytotoxicity is cell-type dependent, as this phenomenon was not observed in THP-1 cells. Alternatively, it could be that CEL interacts with the AmC, reducing its cytotoxicity. 

### 3.5. Inflammatory Response

**Figure 5:**
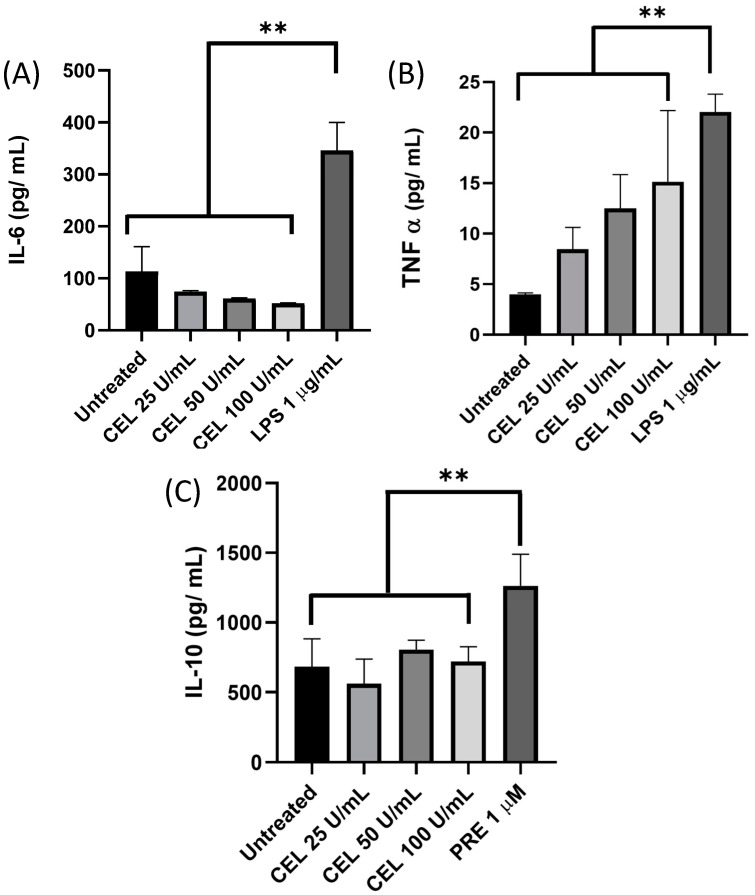
Inflammatory response of CEL. The inflammatory response of CEL was assessed using the THP-1 cell line using ELISA. (**A**) IL-6, (**B**) TNF-α, and (**C**) IL-10. CEL, cellulase; LPS, lipopolysaccharide (positive control); PRE, prednisolone (positive control). Data are shown as the mean ± SD of three independent experiments. ** *p*-value < 0.001.

The analysis of the inflammatory response revealed that the CEL treatment showed no significant increase in the secretion of the pro-inflammatory cytokines IL-6 and TNF-α compared with the lipopolysaccharide control (Figure 5A,B). Similar results were obtained when the anti-inflammatory cytokine IL-10 was assessed (Figure 5C).

## 4. Discussion

Necrotic immature teeth have a high prevalence of strict and facultative anaerobic and facultative aerobic microorganisms. *E. faecalis* is frequently recovered from persistent secondary infections, which are associated with failures of root canal treatments. These persistent infections may derive from the invasion of the periradicular tissue, with the subsequent development of abscesses [11,29]. *E. faecalis* is characterized by its high resistance to standard treatments due to its adaptability and environmental resistance. This adaptability includes the development of copious amounts of biofilm [18], as evidenced by the synthesis of EPS at the beginning of the invasive stage [15].

In the present study, a panel of clinical isolates of *E. faecalis* was tested on the ability of the isolates to produce EPS. Most strains were categorized as moderate biofilm producers, except for the M4 isolate, which showed a high biofilm production in TSB. This broth is a medium rich in peptides (20 mg/mL) and carbohydrates (2.5 mg/mL glucose), favoring *E. faecalis* growth compared with the brain and heart infusion broth (lower glucose concentration) [30]. These results correlate with those of another publication that studied similar oral strains, in which 74% of the strains were described as moderate and high biofilm producers [28]. 

The FTIR analysis of the EPS produced by M4 confirmed the presence of the β (1–4) glycosidic bond (C-O-C stretching), which was previously reported [31]. 

The present study tested biofilm disruption using a commercial CEL composed of three enzymes, namely an endo-1,4-β-glucanase (hydrolysis of random bonds), a β-glucosidase that converts cellobiose in glucose, and an exo-1,4-β-glucanase or cellobiohydrolase that attacks the polymer termination. The biofilm disruption results align with previous studies, which showed that these enzymes inhibit biofilm formation, detach established biofilm, and increase the susceptibility of mature biofilms to antimicrobial agents [32]. In addition, other studies reported that other enzymes, such as trypsin and lysozyme, can promote the disintegration of multispecies oral biofilms [33,34].

Interestingly, a transcriptomic study revealed that plasmid replication and genetic modification genes were upregulated in an *Enterococcus* biofilm, suggesting that the biofilm promotes a horizontal gene transfer [35]. Therefore, the importance of the disaggregation of the biofilm matrix could prevent the development of multidrug-resistant strains [36].

Over the last few years, intra-canal preparations containing antibiotic mixtures at concentrations safe for fibroblasts have been studied. These preparations were used to disinfect the root canal during endodontic regeneration procedures to favor root development and improve the treatment prognosis for necrotic immature teeth [37,38,39]. Therefore, an irrigation protocol combining enzymes and an antibiotic mixture has potential as a new canal disinfection treatment. 

It is known that a combination of antibiotics can reduce the probability of developing resistant bacteria. However, it should be noted that the diffusion of antibiotics through the dentin (post-treatment) could lead to non-inhibitory concentrations promoting bacterial resistance, especially when the biofilm is intact and not previously altered or disintegrated [39].

Our study proposes using an antibiotic combination (AmC) which has the advantage of relying on the supplementation of CEL to disrupt biofilms. We showed that this combination improved the antibiofilm action by eradicating 91.4% of the bacteria. This formulation could decrease the need for administering higher antibiotic concentrations (toxic for fibroblasts) or the loss of the antibiotics by leakage through the dentin. Moreover, our study showed a superior behavior to that shown in a report in which 51 single-rooted premolars were inoculated with a reference strain of *E. faecalis*. The premolars were treated with three commercial disinfecting solutions: SilverSol (10 ppm with 0.1% H_2_O_2_), HYBENX Root Canal Cleanser (a mixture of hydroxybenzenesulfonic acid, hydroxymethoxybenzenesulfonic acid, and sulfuric acid), and QMix 2 in 1 (a combination of EDTA, chlorhexidine, and cetrimide). NaOCl was used as a positive control [40]. The authors reported that none of these commercial solutions could disrupt or eliminate the biofilm compared with NaOCl, which showed a 100% depletion of bacteria in the biofilm. In another study, the *E. faecalis* biofilm was not completely removed despite using NaOCl and four different techniques to prepare the root canal [41]. In our study, a bacterial depletion > 90% was observed for the combined treatment (Cel-AmC). Therefore, we envision the enzyme/antibiotics mixture being more effective when combined with mechanical instrumentation or other irrigants, such as NaOCl, at lower concentrations (below 2.25%). This lower concentration would decrease the erosion of the teeth and show reduced toxicity [42,43,44,45]. Moreover, a low concentration of NaOCl used against a mature *E. faecalis* biofilm in a root canal has been reported to significantly improve the disinfection when mechanical instrumentation or irrigation using Navitio FX was used [46].

## 5. Conclusions

The DFR model established in this work could be applied to evaluate new disinfection alternatives in regenerative endodontic therapy. The combination of antimicrobials with enzymes is an efficient alternative for the treatment of biofilm-associated infections. In this study, combining CEL with a common antibiotics mixture resulted in an antibiofilm capacity upon EPS destabilization, with significant bacterial load reduction, lower cytotoxic effects, and no pro-inflammatory response compared with the other treatments tested. Additional studies are necessary to determine the use of different enzymes and their cytotoxicity in combination with other antibiotics or chemical substances. Nevertheless, these new combinations could offer a unique perspective in further efforts to improve the disinfection protocols associated with regenerative endodontics.

## Figures and Tables

**Table 1:** Glucose release from the hydrolysis of cellulose using CEL.

pH	Enzyme Concentration (U/mL)	Glucose Concentration (mg/mL ± SD)
5	10	0.4973 (0.05)
	100	0.7900 (0.03)
7	10	0.3701 (0.04)
	100	0.6168 (0.04)
8	10	0.3499 (0.09)
	100	0.5221 (0.02)

**Table 2:** Evaluation of antibiofilm treatment on microcolony and periapical biofilms.

Treatment	Microcolony Biofilm		Periapical Biofilm	
	CFU Log Reduction	CFU % Reduction	CFU Log Reduction	CFU % Reduction
NaOCl	8.5 (0.2)	100 (0.00)	8.44 (0.36) **	100 (0.00)
CEL	0.49 (0.09)	67.6 (0.16)	0.34 (0.04)	43 (0.2)
AmC	0.58 (0.11)	73.0 (0.50)	0.30 (0.01)	50 (0.88)
CEL-AmC	1.62 (0.11) *	97.6 (0.45)	1.06 (0.02) **	91.4 (0.31)

CEL, cellulase at 100 U/mL; AmC, amoxicillin combined with clavulanic acid at 1000 and 500 µg/mL, respectively; CEL-AmC, CEL at 50 U/mL and AmC at 1000 and 250 µg/mL, respectively. Shown are the mean (±SD). * *p*-value < 0.05 for microcolony assay compared with the control group. ** *p*-value < 0.05 for periapical assay compared with the control group. The statistical analysis was performed using the Kruskal–Wallis test.

## Data Availability

Data are available upon request.

## Supplementary Materials

The following supporting information can be downloaded at: https://www.mdpi.com/article/10.3390/pharmaceutics15031010/s1, Figure S1. Biofilm staining using Alcian Blue. Figure S2. Periapical biofilms after irrigation. Table S1. Antimicrobial susceptibility profile of *E. faecalis* strains by the Kirby-Bauer method (inhibition zone). Table S2. Extended antimicrobial susceptibility profile of selected *E. faecalis* strain (Dilution test, MIC).
