# Supplementary material for: Use of a Cellulase from Trichoderma reesei as an Adjuvant for Enterococcus faecalis Biofilm Disruption in Combination with Antibiotics as an Alternative Treatment in Secondary Endodontic Infection
Source: Pharmaceutics. 2023 Mar 21;15(3):1010. doi: 10.3390/pharmaceutics15031010 (PMC10059093; doi:10.3390/pharmaceutics15031010)
Supplement: Supplementary file 1 [file pharmaceutics-15-01010-s001.zip › pharmaceutics-2269608-supplementary.pdf]

SUPPLEMENTARY INFORMATION

Use of a Cellulase from *Trichoderma reesei* as an Adjuvant for *Enterococcus faecalis* Biofilm Disruption in Combination with Antibiotics as an Alternative Treatment in Secondary Endodontic Infection

Selene Velázquez-Moreno [1], Ana Maria González-Amaro [2], Antonio Aragón-Piña [3], Lluvia Itzel López-López [4], Roberto Sánchez-Sánchez [5], Mario Alberto Pérez-Díaz [5,6], Ricardo Oliva Rodríguez [2], Ana C. Lorenzo-Leal [7], Omar González-Ortega [8,9], Fidel Martinez-Gutierrez [1,9,10,*] and Horacio Bach [7,*]

[1] Microbiology Laboratory, School of Chemical Sciences, Autonomous University of San Luis Potosí, San Luis Potosí 78300, Mexico
[2] Endodontics Postgraduate Program, School of Dentistry, Autonomous University of San Luis Potosí, San Luis Potosí 78300, Mexico
[3] Electronic Microscopy Laboratory, Institute of Metallurgy, Autonomous University of San Luis Potosí, San Luis Potosí 78300, Mexico
[4] Institute of Desert Zones, Autonomous University of San Luis Potosí, San Luis Potosí 78300, Mexico
[5] National Institute of Rehabilitation, Mexico City 14389, Mexico
[6] Biomembranes Laboratory, National School of Biological Sciences, National Polytechnic Institute, Mexico City 07738, Mexico
[7] Division of Infectious Diseases, Department of Medicine, University of British Columbia, Vancouver, BC V6T 1Z4, Canada
[8] Bioseparations Laboratory, School of Chemical Sciences, Autonomous University of San Luis Potosí, San Luis Potosí 78300, Mexico
[9] Center for Research in Health Sciences and Biomedicine, Autonomous University of San Luis Potosí, San Luis Potosí 78300, Mexico
[10] Laboratorio de Antimicrobianos Biopelículas y Microbiota, Facultad de Ciencias Químicas, Autonomous University of San Luis Potosí, San Luis Potosí 78210, Mexico
* Correspondence: fidelmicro@gmail.com (F.M.-G.); hbach@mail.ubc.ca (H.B.); Tel.: +1-604-8754111 (ext. 62107) (H.B.)

Table S1. Antimicrobial susceptibility profile of *E. faecalis* strains by the Kirby-Bauer method (inhibition zone)[a]

Antibiotic	CLSI Reference (mm)			*E. faecalis* strains																		
	R	I	S	E.F M1	E.F M2	E.F M3	E.F M4	E.F M5	E.F M6	E.F M7	E.F M8	E.F M9	E.F M10	E.F M11	E.F M12	E.F M13	E.F M14	E.F M15	E.F M16	E.F M17	E.F M18	E.F M19
**Amoxicillin/Clavulanate 20/10 µg**	≤16		≥17	S	S	S	S	S	S	S	S	S	S	S	S	S	S	S	S	S	S	S
**Clindamycin 2 µg**	≤14	15-20	≥21	R	R	R	R	R	R	R	R	R	R	R	R	R	R	R	R	R	R	R
**Cephalothin 30 µg**	≤14	15-17	≥18	R	I	R	R	R	R	R	R	R	R	R	R	R	R	R	R	R	R	R
**Vancomycin 30 µg**	≤14	15-16	≥17	S	S	S	S	S	S	S	S	S	S	S	S	S	S	S	S	S	S	S

S= Sensitive, I= Intermedius, R= Resistant.
a=According to CLSI (2021)

Table S2. Extended antimicrobial susceptibility profile of selected *E. faecalis* strain (Dilution test, MIC) [a]

Antibiotic	CLSI Reference (μg/mL)			Susceptibility profile of M4
	Sensitive	Intermedium	Resistant	
**Ampicillin**	≤8		≥16	S
**High level Gentamicin**	≤16	15-16	≥8	S
**Ciprofloxacin**	≤1	2	≥4	S
**Levofloxacin**	≤2	4	≥8	S
**Erythromycin**	≤0.5	1-4	≥8	R
**Linezolid**	≤2	4	≥8	S
**Vancomycin**	≤4	8-16	≥32	S
**Doxycycline**	≤4	8	≥16	R
**Tetracycline**	≤4	8	≥16	R
**Nitrofurantoin**	≤32	64	≥128	S

S= Sensitive, I= Intermedius, R= Resistant.

a=According to CLSI (2021)

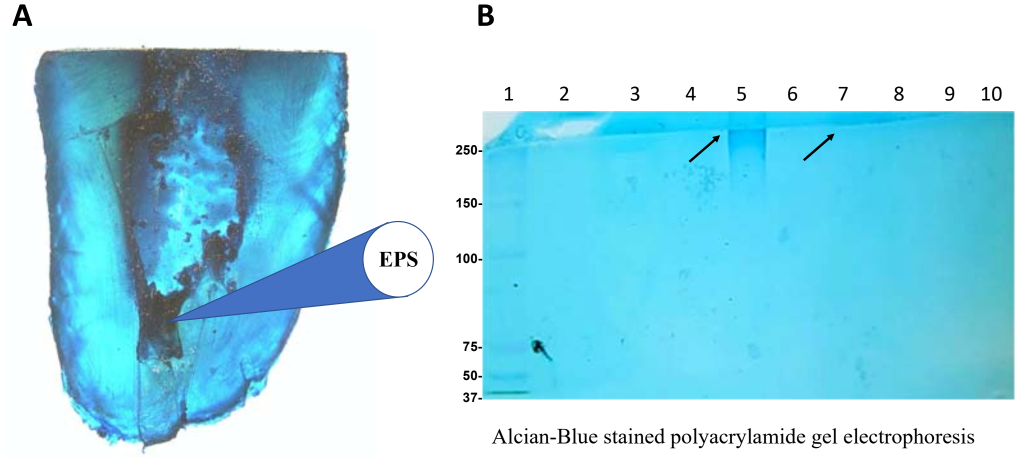

Figure S1. Biofilm staining using Alcian Blue. (**A**) Periapical stained biofilm of *E. faecalis* shown in an infected tooth. (**B**) Biofilm produced by the clinical isolate of *E. faecalis* and separated in SDS-PAGE (10%). Lanes: 1, Pre-stained protein marker; 3, Hydrolase alone; 5, Untreated biofilm; 7 Treated biofilm with the hydrolase. Equal amounts of the biofilm and hydrolase were loaded. Black arrows point to the band of the untreated (Lane 5) and treated biofilm (Lane 7).

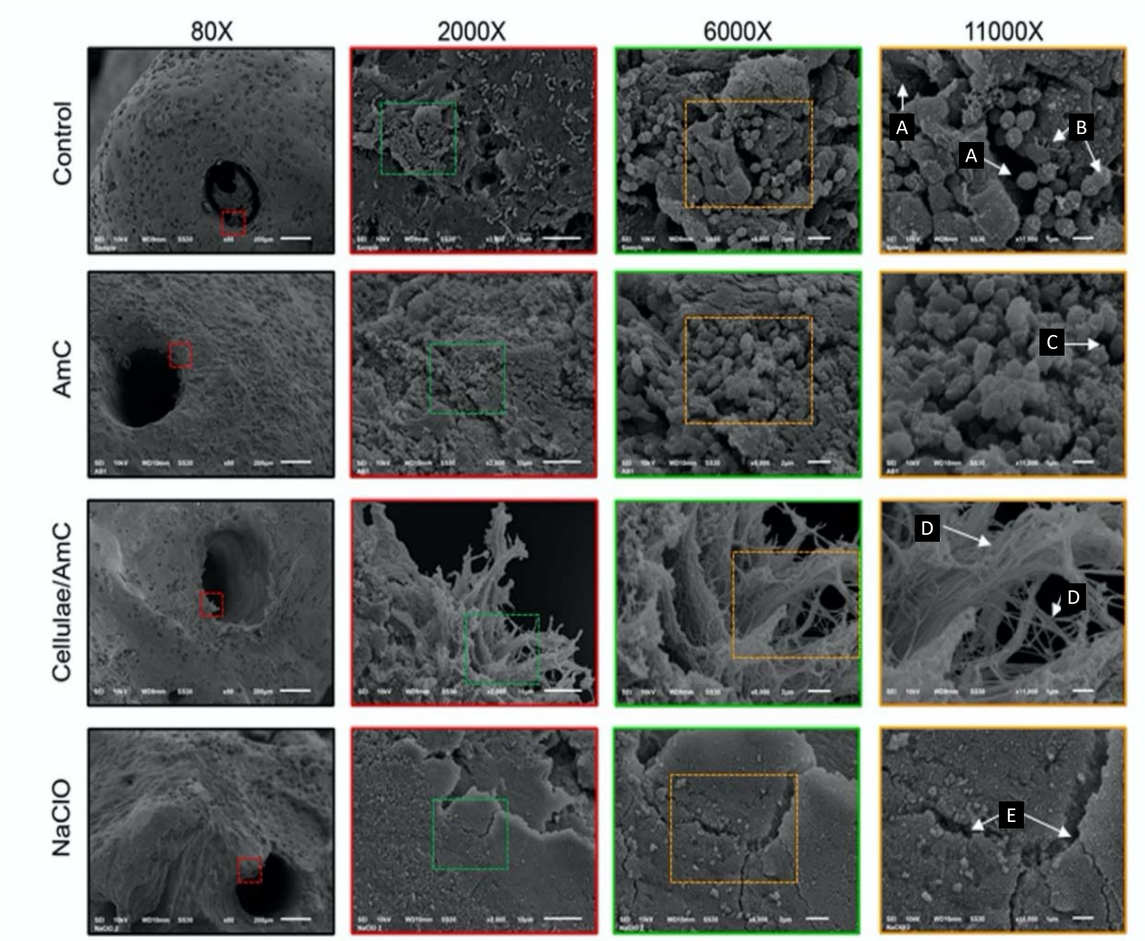

Figure S2. Periapical biofilms after irrigation. The arrangement of images shows the biofilms by treatment group in rows. The increasing order from left to right can be observed in the magnifications of each group. The color boxes show the amplification zones in the micrograph on the right. The last column shows the greatest magnification, with signs in the structure. AmC, Ampicillin and clavulanic acid. A) and C) Possible communication pores, B) EPS excreted by the bacteria, D) Possible fibers resulting from the hydrolysis of EPS polymers by CEL, E) Possible microfractures in dentin.
